# Fully automatic categorical analysis of striatal subregions in dopamine transporter SPECT using a convolutional neural network

**DOI:** 10.1007/s12149-025-02038-3

**Published:** 2025-03-16

**Authors:** Thomas Buddenkotte, Catharina Lange, Susanne Klutmann, Ivayla Apostolova, Ralph Buchert

**Affiliations:** 1 Department of Diagnostic and Interventional Radiology and Nuclear Medicine, University Medical Center Hamburg-Eppendorf, Hamburg, Germany; 2 Department of Nuclear Medicine, Charité, Universitätsmedizin Berlin, Corporate Member of Freie Universität Berlin and Humboldt-Universität zu Berlin, Berlin, Germany

**Keywords:** Deep learning, Convolutional neural network, Dopamine transporter, SPECT, FP-CIT

## Abstract

**Objective:**

To provide fully automatic scanner-independent 5-level categorization of the [^123^I]FP-CIT uptake in striatal subregions in dopamine transporter SPECT.

**Methods:**

A total of 3500 [^123^I]FP-CIT SPECT scans from two in house (*n* = 1740, *n* = 640) and two external (*n* = 645, *n* = 475) datasets were used for this study. A convolutional neural network (CNN) was trained for the categorization of the [^123^I]FP-CIT uptake in unilateral caudate and putamen in both hemispheres according to 5 levels: normal, borderline, moderate reduction, strong reduction, almost missing. Reference standard labels for the network training were created automatically by fitting a Gaussian mixture model to histograms of the specific [^123^I]FP-CIT binding ratio, separately for caudate and putamen and separately for each dataset. The CNN was trained on a mixed-scanner subsample (*n* = 1957) and tested on one independent identically distributed (IID, *n* = 1068) and one out-of-distribution (OOD, *n* = 475) test dataset.

**Results:**

The accuracy of the CNN for the 5-level prediction of the [^123^I]FP-CIT uptake in caudate/putamen was 80.1/78.0% in the IID test dataset and 78.1/76.5% in the OOD test dataset. All 4 regional 5-level predictions were correct in 54.3/52.6% of the cases in the IID/OOD test dataset. A global binary score automatically derived from the regional 5-scores achieved 97.4/96.2% accuracy for automatic classification of the scans as normal or reduced relative to visual expert read as reference standard.

**Conclusions:**

Automatic scanner-independent 5-level categorization of the [^123^I]FP-CIT uptake in striatal subregions by a CNN model is feasible with clinically useful accuracy.

**Supplementary Information:**

The online version contains supplementary material available at 10.1007/s12149-025-02038-3.

## Introduction

Single-photon emission computed tomography (SPECT) with the dopamine transporter (DAT) ligand N-ω-fluoropropyl-2β-carbomethoxy-3β-(4-I-123-iodophenyl)nortropane ([^123^I]FP-CIT) is widely used for the detection (or exclusion) of nigrostriatal degeneration in patients with a clinically uncertain Parkinsonian syndrome or suspected dementia with Lewy bodies [1–5]. In clinical practice, the interpretation of DAT-SPECT images is primarily dichotomous as either “reduced” striatal tracer uptake (indicative of nigrostriatal degeneration) or “normal” striatal uptake (indicative of secondary parkinsonism not associated with nigrostriatal degeneration). However, the spatial pattern of the reduction across striatal subregions also provides important information. In Parkinson’s disease (PD), the reduction is usually more pronounced in the putamen compared with the caudate, and it is more pronounced in the brain hemisphere contralateral to the body side that is more strongly affected by the neurologic symptoms [6]. In dementia with Lewy bodies, early involvement of the caudate can result in a rather uniform loss of the striatal signal, that is, without the steep rostro-caudal gradient often seen in PD [7]. Corticobasal degeneration can present with greater hemispheric asymmetry of the striatal loss compared to PD [8]. Furthermore, “atypical” patterns caused by structural/vascular pathology should be recognized in order to avoid misinterpretation [9].

The EANM/SNMMI practice guideline for dopaminergic imaging in Parkinsonian syndromes states that “visual assessment is usually sufficient for evaluating striatal left/right symmetry and striatal subregions”. But the guideline also acknowledges that semi-quantitative analysis can be a useful “adjunct to visual interpretation” [4]. This is supported by a study of Booij and co-workers demonstrating that the diagnostic performance of visual reading of [^123^I]FP-CIT SPECT with the support of conventional semi-quantitative analysis is not inferior to visual reading alone and offers an increase in reader confidence [10].

The most widely used semi-quantitative measure in both research and clinical practice is the specific binding ratio (SBR) of [^123^I]FP-CIT [11]. Region-of-interest-based techniques can be used to estimate the SBR for unilateral striata and striatal subregions such as putamen and caudate. From these, the asymmetry between left and right putamen or caudate SBR can be computed to characterize hemispheric asymmetry, the putamen-to-caudate SBR ratio can be computed to characterize the rostro-caudal gradient. A major disadvantage of these SBR-based analyses is their sensitivity with respect to site- and/or camera-specific variability of SPECT hardware and reconstruction software [12–16]. This limits the sharing of normative reference databases and cutoff values on SBR-based measures between sites and cameras. Furthermore, SBR-based measures are also affected by scan-specific variability including head motion and rotation radius of the detector heads [17, 18].

Against this background, the aim of the current study was to design, train and validate a deep learning-based approach for regional categorization of the specific [^123^I]FP-CIT binding using a 5-level score (normal, borderline, moderate reduction, strong reduction, almost missing) that is less sensitive to camera- and site-specific variability than SBR-based measures but still allows discrimination between normal, PD-like and atypical striatal uptake patterns. The rationale for using a 5-level scheme was to reach a compromise between the utility of the categorization for semi-quantitative characterization of the specific [^123^I]FP-CIT binding (better with more levels) and the reliability of the automatic categorization (better with less levels). A total of 4 different datasets comprising a total of 3500 different [^123^I]FP-CIT SPECT scans were used for this purpose.

Previous studies on the use of deep learning to support the interpretation of DAT-SPECT focused on the dichotomous classification of the whole scan [19–43].

## Materials and methods

### Datasets

Four datasets, 2 in-house datasets and 2 external datasets, with a total of 3500 different [^123^I]FP-CIT-SPECT scans were included retrospectively.

The first in-house dataset comprised 1740 consecutive clinical [^123^I]FP-CIT-SPECT scans acquired between 12/2008 and 01/2020 with four different cameras [44, 45]: Siemens e.cam dual-head camera equipped with low-energy-high-resolution collimators, Siemens Symbia TruePoint dual-head camera with low-energy-high-resolution collimators, Siemens Symbia TruePoint with fan-beam collimators, and Mediso AnyScan Trio triple-head camera equipped with low-energy-high-resolution-high-sensitivity collimators in dual-head mode. Detailed acquisition parameters are given in Supplementary Table 1. Consistent image reconstruction was performed retrospectively using the iterative ordered-subsets-expectation–maximization algorithm with attenuation and simulation-based scatter correction as well as collimator–detector response modeling implemented in the Hybrid Recon-Neurology tool of the Hermes SMART workstation v1.6 (5 iterations, 15/16 subsets for 120/128 views, 7 mm Gaussian postfiltering). For a priori data augmentation, each scan was reconstructed a second time using the same reconstruction algorithm but without correction for photon attenuation and scatter. This resulted in a total of 3480 SPECT images. The binary gold-standard label on scan level as either “normal” or neurodegeneration-typical reduction of the striatal signal was obtained by visual interpretation of the DAT-SPECT images by 3 independent readers (majority vote) [44].

The second in-house dataset comprised 640 consecutive clinical [^123^I]FP-CIT-SPECT scans acquired between 01/2020 and 05/2022 using a Mediso AnyScan Trio triple-head camera equipped with second-generation general purpose brain multiple-pinhole collimators. The latter provide high-count sensitivity at the center of the field-of-view with a rather broad peak of the sensitivity profile for improved stability with respect to off-center positioning [46, 47]. The energy window was set to 143–175 keV. The distance between the center-of-rotation axis and the pinhole focal plane was fixed at 140 mm. Helical acquisition mode with 40 mm total table displacement was used to avoid axial undersampling [48, 49]. A total of 90 projection views (30 per head, 120° scan arc) at angular steps of 4° were acquired in list mode. Projection data were sorted into 256 × 256 matrices with 2.13 × 2.13 mm^2^ pixel size. The acquisition time per projection was 60 s resulting in 30-min total net scan duration [21]. Transaxial images with cubic voxels of 1.8 mm edge length were reconstructed with the Monte Carlo photon simulation engine and iterative one-step-late maximum-a-posteriori expectation–maximization implemented in the camera software (24 iterations, 2 subsets) [21, 47, 50]. Correction for photon attenuation and scatter was not performed [51]. For a priori data augmentation, each scan was reconstructed 3 more times using the same reconstruction algorithm but restricting the events in the raw projection list mode data to reduced scan duration of 20, 15 and 12 min [21]. This resulted in a total of 2560 SPECT images. The binary gold standard label on scan level was obtained by visual interpretation by an experienced reader [21].

The first external dataset comprised 645 [^123^I]FP-CIT-SPECT from the Parkinson’s Progression Markers Initiative (PPMI) (www.ppmi-info.org/data) [42]. The clinical diagnosis was used as gold-standard label on scan level (Parkinson’s disease = “reduced “, healthy control = “normal “). Details of the PPMI DAT-SPECT protocol are given at http://www.ppmi-info.org/study-design/research-documents-and-sops/.

The second external dataset comprised 475 consecutive clinical [^123^I]FP-CIT-SPECT scans from the Charité university medical center, all acquired with the same GE Discovery NM/CT 670 Pro dual-head camera equipped with low-energy-high-resolution collimators. The energy window was set to 143–175 keV. The mean radius of rotation was 151 ± 16 mm. A total of 120 projection views (60 per head, 180° scan arc) at angular steps of 3° were acquired. Projections were sorted into 128 × 128 matrices with 3.32 × 3.32 mm^2^ pixel size. The acquisition time per projection was 30 s resulting in 30 min total net scan duration. The SPECT images were reconstructed with DaTQUANT v1.0 on a Xeleris workstation (GE Healthcare) using the OSEM algorithm with default parameters (2 iterations, 10 subsets for 128 views, Butterworth filter with cutoff of 0.6 cycles/cm and power of 10, Chang attenuation and scatter correction). The binary gold-standard label on scan level was obtained by visual interpretation by an experienced reader.

More details on the datasets are given in Table 1. Ten randomly selected images from each of the 4 datasets are shown in Supplementary Fig. 1. There were obvious differences between the datasets with respect to spatial resolution and the level of statistical noise. This is despite the fact that all SPECT images included in this study had been acquired and reconstructed in line with common procedures guidelines [52, 53].

**Table 1:** Datasets

	Internal 1 (LEHR, fan beam)	Internal 2 (multiple pinhole)	External 1 (PPMI)	External 2 (Charité)
Use	Training (2/3) and testing (1/3, IID)	Training (2/3) and testing (1/3, IID)	Training (2/3) and testing (1/3, IID)	Testing only (OOD)
Number of scans	1740	640	645	475
Age (y)	66.7 ± 11.6 (20–90)	67.2 ± 11.4 (26–91)	61.2 ± 10.2 (30–84)	69.1 ± 12.2 (14–89)
Females (%)	43.5	44.2	35.2	42.7
Binary reference standard (“reduced” versus “normal”)	Majority vote of 3 readers	Single reader	Clinical diagnosis	Single reader
% reduced	48.1	51.1	67.9	46.5
A priori data augmentation	With and without attenuation and scatter correction	Reconstruction of 12, 15, 20, 30-min raw data	None	None
Total number of images	3480	2560	645	475

### Image preprocessing

Preprocessing of the DAT-SPECT images was performed as described previously [21]. In brief, individual [^123^I]FP-CIT-SPECT images were spatially normalized to the Montreal Neurological Institute space using the Normalize tool of the Statistical Parametric Mapping software package (SPM12) and a set of custom [^123^I]FP-CIT-SPECT template images (representative of normal striatal signal and different levels of PD-like reductions) [45]. Semi-quantitative distribution volume ratio (DVR) images were obtained by voxelwise scaling to the individual 75th percentile of the voxel intensity in a reference region comprising the whole brain without striata, thalamus, medial temporal lobe, brainstem, cerebellum, and ventricles [54].

### Regional specific binding ratios

Unilateral [^123^I]FP-CIT SBR of left and right caudate and left and right putamen were obtained by hottest voxels analysis of the spatially normalized DVR image using large unilateral masks predefined in template space [42]. These masks were much bigger than caudate and putamen in order to guarantee that the entire anatomical structures were fully included in the masks in each individual patient, independent of some residual anatomical between-subjects variability after spatial normalization. The number of hottest voxels over which to average the DVR was fixed to 5 and 10 ml (625 and 1250 cubic voxels of 2 mm edge length), in line with the volumes of unilateral caudate and putamen in healthy subjects [55]. The SBR was computed as SBR = DVR–1.

### 5-score for the categorical characterization of unilateral putamen and caudate

The following 5 levels were used for the categorical characterization of the [^123^I]FP-CIT uptake in striatal subregions: 0 = “normal”, 1 = “borderline”, 2 = “moderate reduction”, 3 = “strong reduction” and 4 = “(almost) missing”. The same levels are used for visual scoring of the subregional [^123^I]FP-CIT uptake in clinical routine at our site.

### Reference standard for the regional 5-score

In order to avoid within- and between-raters variability associated with visual scoring of striatal subregions, a fully automatic method was employed to generate reference standard labels for the regional 5-level score. This was done separately for each of the 4 datasets and separately for putamen and caudate. It was done independent of the hemisphere, that is, SBR estimates from left and right hemisphere were gathered together into a single sample (doubling the sample size).

First, the distribution of the SBR, characterized by a histogram with 0.1 bin width, was fitted by the following Gaussian mixture model [44]:

1 $$histogram\,\left(SBR\right)={A}_{1}exp\left(-\frac{{\left(SBR-{M}_{1}\right)}^{2}}{{2SD}_{1}^{2}}\right)+{A}_{2}exp\left(-\frac{{\left(SBR-{M}_{2}\right)}^{2}}{{2SD}_{2}^{2}}\right),$$

where* A*_*1*_, *A*_*2*_ are the amplitudes, *M*_*1*_, *M*_*2*_ are the mean values and *SD*_*1*_, *SD*_*2*_ are the standard deviations of the Gaussian functions. The MATLAB routine “fminsearch” with default parameter settings was used for this purpose. The cutoff *c*_*dicho*_ for dichotomization of the SBR as “reduced” or “non-reduced” was selected halfway between *M*_*1*_ and *M*_*2*_ in units of standard deviations, that is.

2 $${c}_{dicho}=\left({{SD}_{2}M}_{1}+{{SD}_{1}M}_{2}\right)/\left({SD}_{1}+{SD}_{2}\right).$$

From the Gaussian mixture model, the reference standard for the regional 5-level score was generated as follows:

0 = “normal”: SBR ≥ 10th percentile of “non-reduced” SBR

1 = “borderline”: *c*_*dicho*_ ≤ SBR < 10th percentile of “normal” SBR

2 = “moderate reduction”: 66th percentile of “reduced” SBR ≤ SBR < *c*_*dicho*_

3 = “strong reduction”: 33th percentile of “reduced” SBR ≤ SBR < 66th percentile of “reduced” SBR

4 = “(almost) missing”: SBR < 33th percentile of “reduced” SBR

Thus, the cutoff *c*_*dicho*_ derived from the Gaussian mixture model was used as “anchor” for the reference standard to separate the 3 “reduced”-categories (moderate reduction, strong reduction, almost missing) from the 2 “non-reduced” categories (borderline, normal). Previous studies have demonstrated that the cutoff *c*_*dicho*_ on the putamen SBR provides high accuracy for the automatic dichotomous discrimination between “reduced” and “non-reduced” scans with visual expert interpretation as reference standard [21, 44].

The rationale for using the 10th percentile of the “non-reduced” SBR as threshold between the “borderline” and the “normal” category was that the proportion of uncertain borderline cases in clinical [^123^I]FP-CIT-SPECT has been reported to be up to 10% [56, 57].

The 66th percentile and the 33th percentile of the “reduced” SBR were used to discriminate between the 3 different “reduced” categories in order to include about the same number of cases into each of these categories.

### Training and test datasets

A heterogeneous mixed-scanner training dataset was generated by randomly selecting 2/3 of the images from each of the two in-house datasets and from the first external dataset. The resulting dataset (4338 images from 1957 different scans) was further split in a fivefold cross-validation manner for CNN training and validation (but not for testing).

The remaining 1/3 of the three aforementioned datasets were merged to the independent identical distribution (IID) test dataset (2347 images from 1068 different scans). The second external dataset served as out-of-distribution (OOD) test dataset.

All images from the same scan were randomized into the same dataset, either the training dataset or the test dataset.

### Convolutional neural network

A 12 mm thick 2-dimensional DVR slab image was obtained from the 3-dimensional DVR images by averaging six 2 mm thick transversal slices through the striatum [58]. A quadratic DVR matrix with 72 rows and 72 columns centered at the striata was cropped from the slab and served as input to the CNN. The DVR values were z-transformed using the global mean and the global standard deviation across all 72 × 72 pixels in all scans in the training dataset. Global mean and global standard deviation computed in the training dataset were also applied for z-transformation of the test datasets.

**Fig. 1:**
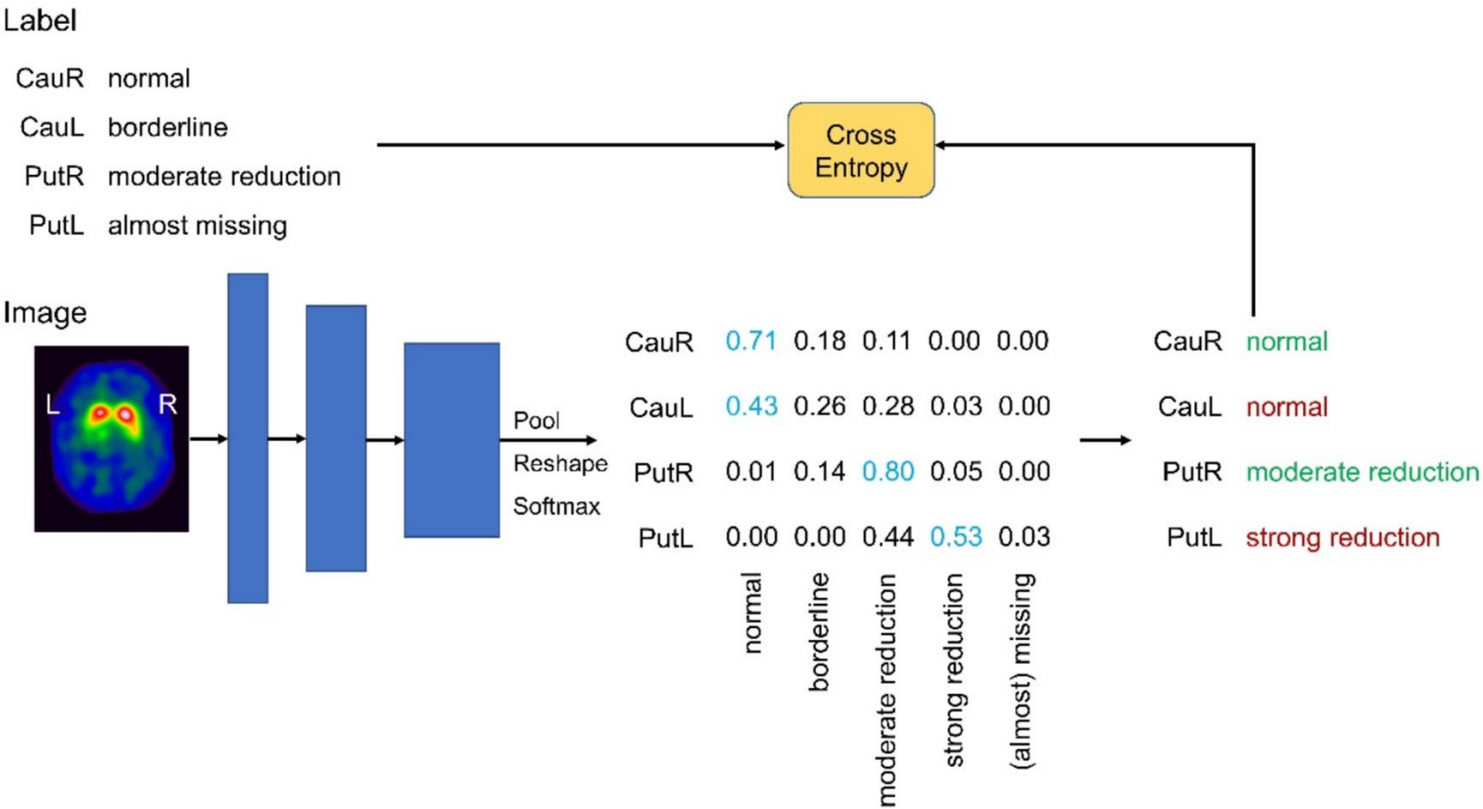
Network architecture and training workflow. The network uses a ResNet-style body and applies spatial pooling, reshaping to a 4 × 5 matrix and row-wise softmax

A custom ResNet CNN architecture was used with three stages, 1, 1, 2 blocks and 16, 32, 64 filters per stage (Fig. 1). Each block was arranged as two stacks of convolution-batch normalization-LReLU followed by the residual connection. The pooling ratio per stage was set to 3. The final block was followed by mean pooling. The resulting feature vector was used as input to four separate linear layers resulting in four output vectors of length 5 (one vector component for each of the 5 categories). Finally, the softmax function was applied separately to each vector component.

The CNN was trained for 50.000 iterations with batch size 12 using a stochastic gradient descent optimizer with Nesterov momentum of 0.9. The learning rate schedule was set to a linear warm-up plus cosine decay with maximum 0.005. The weight decay was fixed to 3e-5. Cross-entropy was used as loss function. Data augmentation on the fly for improved robustness of the trained CNN included random grid rotation and scaling, as well as Gaussian blurring and additive random noise as described previously [59].

The training was performed in a fivefold cross-validation manner. Hyper-parameters were tuned to maximize the fivefold cross-validation performance.

The inference in the test datasets (IID, OOD) was carried out by evaluating all five networks obtained in cross-validation and averaging the softmax outputs.

### Statistical analysis

The performance of the CNN ensemble for the prediction of the regional 5-level score was assessed on the regional level, separately for putamen and caudate, and on the patient level. The CNN prediction was considered correct on the patient level if the predicted 5-score was correct for each of the 4 subregions.

The region-level accuracy (in %) of the CNN for the prediction of the category of the putaminal [^123^I]FP-CIT signal was computed as 100 * number of correctly categorized putamina across all test cases, both hemispheres and all categories/total number of putamina in the test dataset. This was performed separately for both test datasets. The region-level accuracy for the prediction of the caudate category was computed analogously.

The region-level sensitivity (in %) of the CNN to identify a given category (normal, borderline, moderate reduction, strong reduction or almost missing) was computed as 100 * number of correctly identified test cases of the considered category/total number of test cases of the considered category. This was performed separately for each of the 5 categories, both striatal subregions and both test datasets.

The scan-level accuracy (in %) of the CNN for the prediction of the 5-level score for all 4 striatal subregions in a given scan was computed as 100 * number of test scans with correct 5-level prediction for all striatal subregions/total number of scans in the test dataset. This was performed separately for both test datasets.

In order to test the utility of the predicted regional 5-level scores for binary categorization of the entire scan, a binary global score was derived from the 4 regional 5-scores. The binary global score was set to “reduced”, if the predicted putamen 5-score indicated “moderate reduction” or worse in at least one hemisphere. The binary global score was set to “normal” in all other cases. The binary scan-level accuracy (in %) of the CNN for the correct classification of the entire scan as “reduced” or “normal” relative to the binary gold-standard label (based on visual expert reading or the clinical diagnosis) was computed as 100 * number of test scans with correct binary classification according to the dichotomized 5-level prediction for all striatal subregions/total number of scans in the test dataset. This was performed separately for both test datasets.

## Results

**Fig. 2:**
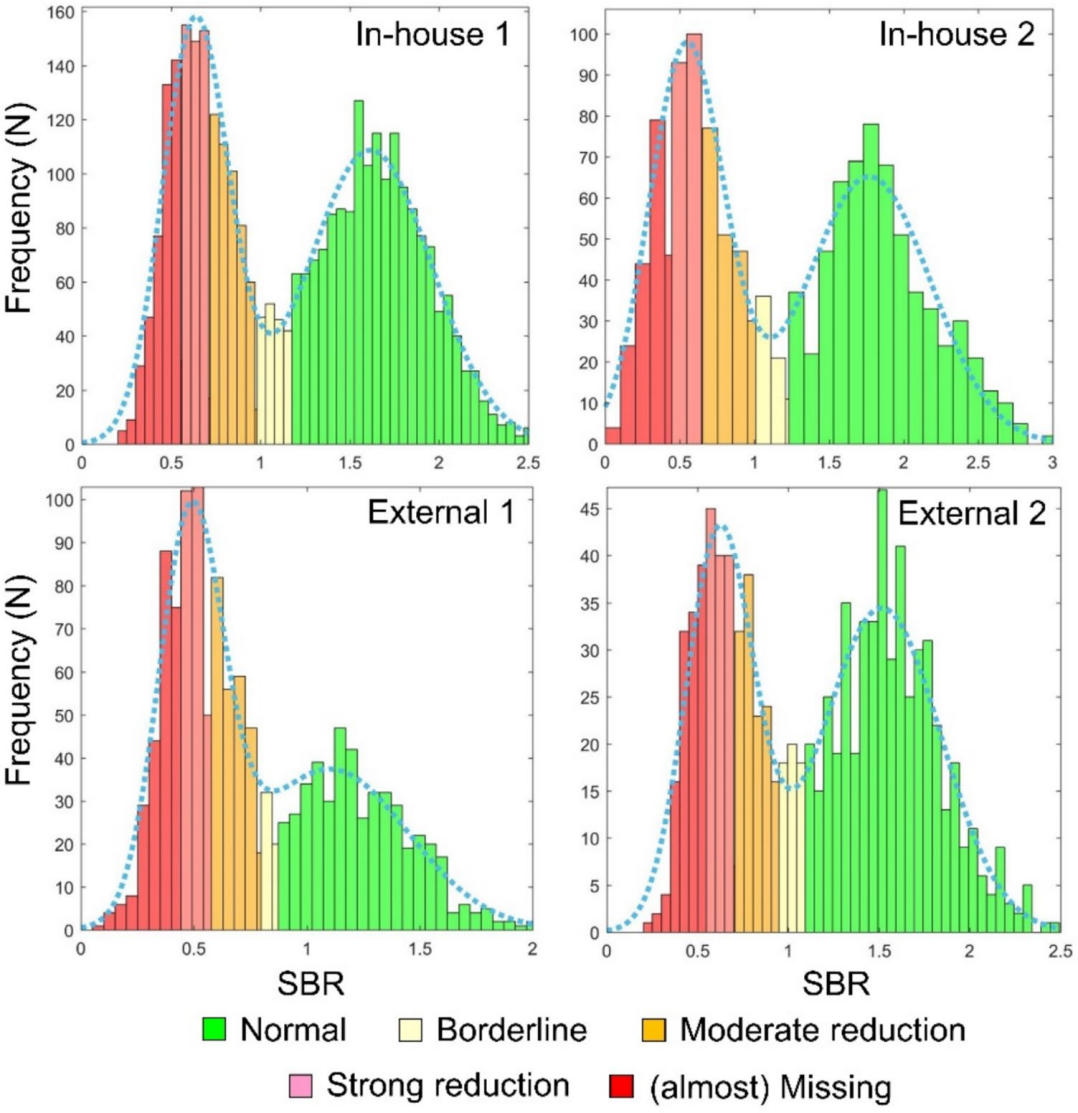
Automatic generation of the reference standard for the categorical 5-level score in the putamen based on the fit of a Gaussian mixture model to the histogram of the putamen specific binding ratio (SBR). This was performed separately for each dataset

The results of the automatic generation of the reference standard for the regional 5-level scores are summarized in Fig. 2 (putamen), Supplementary Table 2 and Supplementary Fig. 2 (caudate). The fit of the SBR histogram by the Gaussian mixture model specified in Eq. (1) worked properly according to visual inspection for the putamen (Fig. 2) as well as for the caudate (Supplementary Fig. 2) in each of the four different datasets, including the first external dataset that was rather imbalanced with respect to the global binary reference standard (67.9% reduced cases, Table 1). Thus, the sample sizes of the datasets were sufficiently large to reliably fit the Gaussian mixture model. The “borderline” cases were located in the dip between the two Gaussians, supporting the thresholds selected for the generation of the regional 5-level score. The SBR values were strongly different between the datasets, as was to be expected from the large differences between the datasets with respect to spatial resolution (Supplementary Fig. 1). For example, the mean putamen SBR of the Gaussian representing non-reduced cases was 2.829 in the second in-house dataset versus 1.691 in the first external dataset. This underlines the need for fitting the Gaussian mixture model separately for each dataset.

**Fig. 3:**
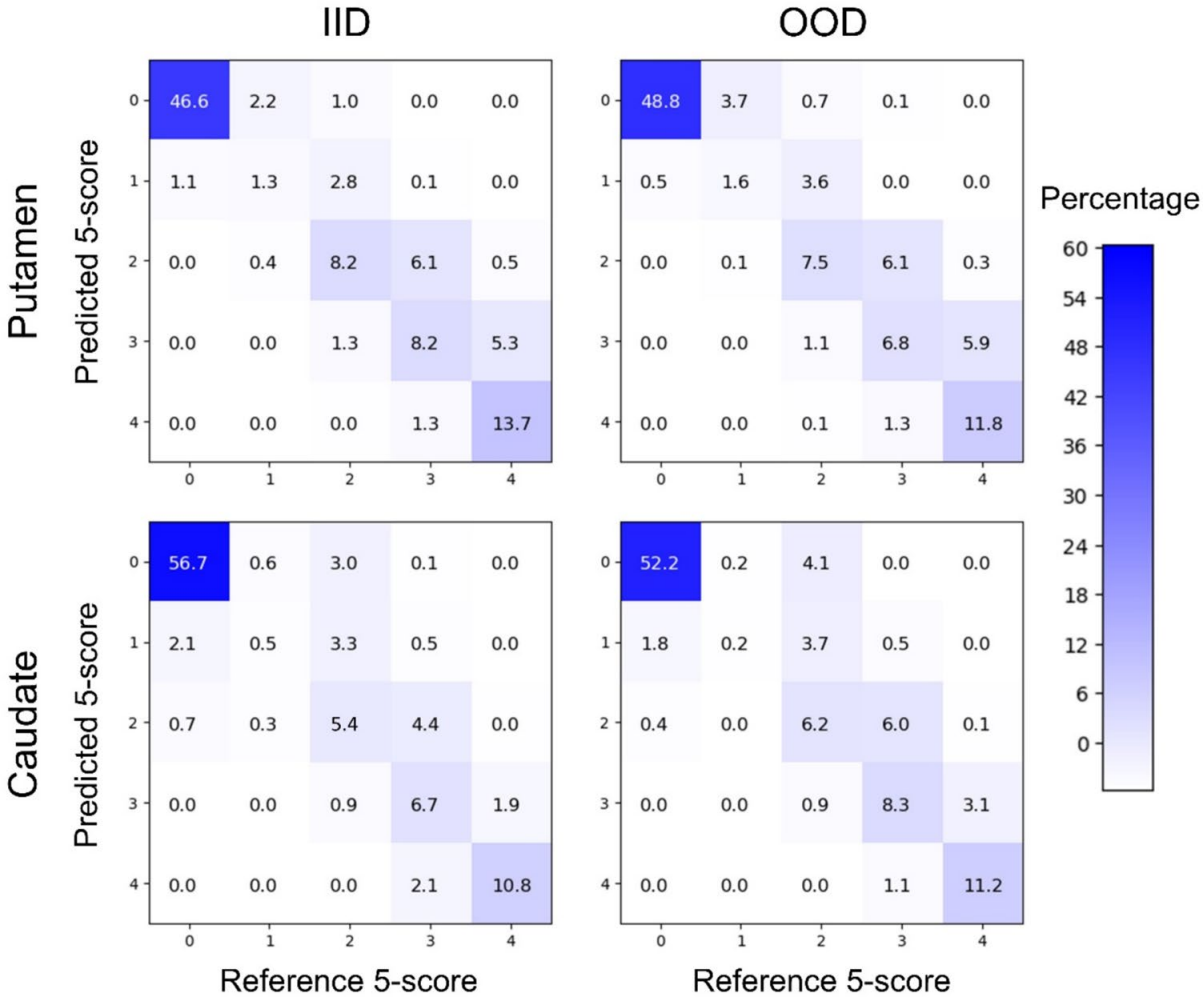
Confusion matrix of the 5-level score between model prediction and reference standard, separately for putamen and caudate and separately for the two independent test datasets: independent identical distribution (IID) and out-of-distribution (OOD) test dataset. In each confusion matrix, the values given in the cells indicate the percentage of cases with the corresponding combination of model prediction versus reference standard relative to the total number of cases in the considered test dataset (IID test dataset: 2347, OOD test dataset: 475). Thus, the values across all cells in the confusion matrix add up to 100, separately for each confusion matrix. (0 = normal, 1 = borderline, 2 = moderate reduction, 3 = strong reduction, 4 = (almost) missing)

The confusion matrix of the CNN-based prediction of the regional 5-level score versus the reference standard is shown in Fig. 3, separately for putamen and caudate and separately for both test datasets (IID, OOD). The CNN-based prediction agreed with the reference standard in 76.5–80.1% of the cases (putamen IID/OOD: 78.0%/76.5%, caudate IID/OOD: 80.1%/78.1%; sum of the percentages in the diagonal cells of the confusion matrix: “prediction = reference”). In most of the discrepant cases, the CNN-based prediction was to a direct neighbor of the reference standard 5-level score (putamen IID/OOD: 20.5%/22.3% of all cases, caudate IID/OOD: 15.6%/16.8%; sum of the percentages in the 8 off-diagonal cells adjacent to the diagonal cells, 4 off-diagonal cells below and 4 off-diagonal cells above the diagonal cells: abs (prediction—reference) = 1). A discrepancy of more than one score point occurred less frequently (putamen IID/OOD: 1.6%/1.2% of all cases, caudate IID/OOD: 4.3%/5.1%; sum of the percentages across the remaining 12 off-diagonal cells not directly adjacent to the diagonal cells, 6 off-diagonal cells below and 6 off-diagonal cells above the diagonal cells: abs (prediction—reference) ≥ 2). A discrepancy of more than two score points was rare (putamen IID/OOD: 0.0%/0.1% of all cases, caudate IID/OOD: 0.1%/0.0%; sum of the percentages in the 6 cells furthest from the diagonal, 3 in the lower left corner and 3 in the upper right corner: abs (prediction—reference) ≥ 3). Underestimation of the reference standard by the CNN-based prediction occurred more often than overestimation (putamen IID/OOD: 18.0% versus 4.1%/20.4% versus 3.1% of all cases, caudate IID/OOD: 13.8% versus 6.1%/17.7% versus 4.2%; sum of the percentages in the off-diagonal cells above versus below the diagonal: prediction < reference versus prediction > reference). In all settings, the most frequent discrepancy was underestimation of “strong reduction” (score 3) according to the reference standard as “moderate reduction” (score 2) (putamen IID/OOD: 6.1%/6.1% of all cases, caudate IID/OOD: 4.4%/6.0%).

Estimates of the overall accuracy of the CNN-based regional 5-level prediction are summarized in Table 2. The CNN-based prediction of the 5-level score was correct in all 4 striatal subregions (putamen and caudate in left and right hemisphere) in 54.3% of the cases in the IID test dataset and in 52.6% of the cases in the OOD test dataset. The accuracy of the regional 5-level predictions on dichotomized scan level (“reduced”, if the predicted putamen 5-score indicated “moderate reduction” or worse in at least one hemisphere) was 97.4% in the IID test dataset and 96.2% in the OOD test dataset.

**Table 2:** Accuracy (%) of the CNN ensemble to predict the 5-level score on the regional level (separately for putamen and caudate), on the scan level (correct, if the predicted 5-score was correct for each of the 4 subregions), and on the scan level after dichotomization (“reduced”, if the predicted putamen 5-score indicated “moderate reduction” or worse in at least one hemisphere)

	Independent identical distribution test dataset	Out-of-distribution test dataset
Region-level putamen	78.0	76.5
Region-level caudate	80.1	78.1
Scan-level (all regional 5-scores correct)	54.3	52.6
Scan-level after dichotomization	97.4	96.2

**Fig. 4:**
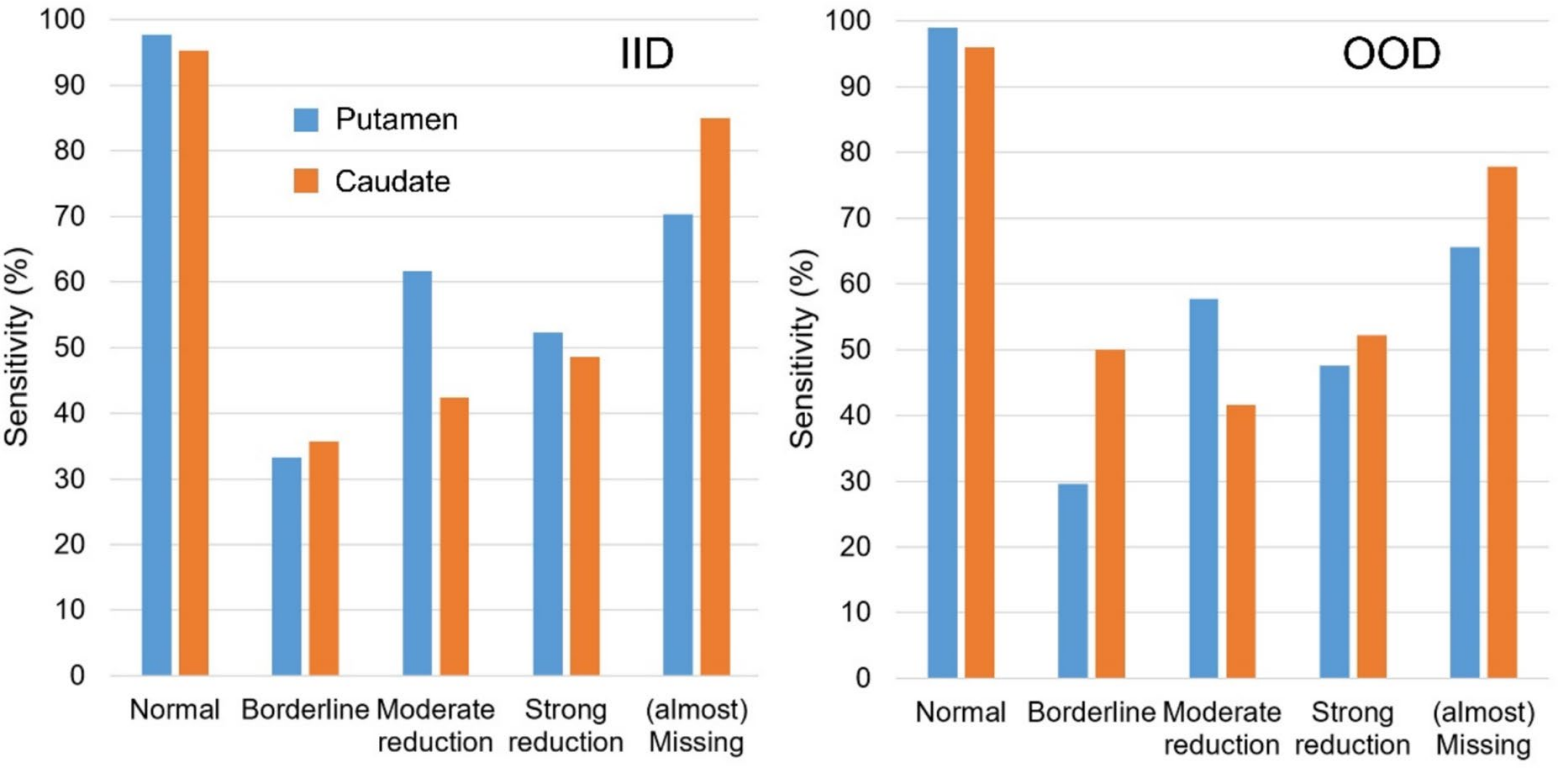
Percentage of cases with correctly predicted 5-level score by the CNN, separately for each of the 5 categories. The results are given for the independent identical distribution (IID) test dataset (left) and for the independent out-of-distribution (OOD) test dataset (right)

Estimates of the sensitivity of the CNN for the prediction of each individual of the 5 different categories are shown in Fig. 4. The sensitivity was highest for the prediction of “normal” [^123^I]FP-CIT uptake in the putamen (IID/OOD: 97.7%/99.0%) as well as in the caudate (IID/OOD: 95.3%/96.0%), followed by the sensitivity for the prediction of “almost missing” [^123^I]FP-CIT uptake (putamen IID/OOD: 70.3%/65.6%, caudate IID/OOD: 85.0%/77.8%). The sensitivity was lowest for the prediction of “borderline” cases, particularly in the putamen (IID/OOD: 33.3%/29.6%).

## Discussion

The aim of this study was to design, train and test a CNN for the 5-level categorization of the [^123^I]FP-CIT uptake in striatal subregions in DAT-SPECT independent of scanner hardware and reconstruction software.

The CNN achieved almost 80% overall accuracy in the prediction of the 5-score on the regional level, more or less independent of the striatal subregion, putamen or caudate (Table 2). The latter is expected to allow reliable differentiation between normal, PD-like and “atypical” caudate-to-putamen gradients. Close to 80% overall accuracy of the regional categorization indicates clinically useful CNN performance, particularly when considering the limitations of the automatically generated reference standard (see limitations section) and that chance level accuracy of predicting a 5-score is only 20% (compared to 50% for prediction of a binary score). Furthermore, the vast majority of the false predictions were to a neighboring category (Fig. 3), which may be without practical relevance in some cases (e.g., “moderate reduction” miscategorized as “strong reduction”). Importantly, overall accuracy in the prediction of the regional 5-score was essentially the same in the OOD test dataset as in the IID test dataset (Table 2). This suggests that the CNN generalizes to data from unseen sources, at least if the image characteristics are within the range covered by the mixed-scanner training data (Supplementary Fig. 1). Strict harmonization of image characteristics most likely is not required.

The CNN-based prediction of the 5-score was correct in all 4 striatal subregions in about 50% of the scans (Table 2). This is in reasonable agreement with about 40% fully correct cases that is expected when the predictions of the 4 striatal subregions of the same scan are independent from each other (0.8 × 0.8 × 0.8 × 0.8 = 0.41). This finding further supports that the CNN-based regional categorization is rather robust against site- and/or camera-specific variability of image characteristics. Site-/camera-specific variability affects all striatal subregions in a similar way, which is expected to cause systematic miscategorization of all striatal subregions of a given scan if the CNN-based prediction is sensitive to site-/camera-specific variability.

The sensitivity of the CNN to identify a given individual category was the largest for the extreme categories (normal, almost missing), it was intermediate for both categories representing incomplete signal reduction (moderate reduction, strong reduction), and it was the smallest for the “borderline” category (Fig. 4). A large proportion of the “borderline” cases was miscategorized as “normal”, most pronounced in the putamen (Fig. 3). This was to be expected from the definition of the “borderline” category as the lowest 10% of the “non-reduced” cases according to the Gaussian mixture model. Miscategorization of “borderline” cases as “normal” does not affect the CNN’s power to identify reduced cases in clinical practice. Lower accuracy for the identification of incomplete signal reduction (moderate reduction, strong reduction) limits the CNN’s utility for the automatic identification of “atypical” patterns and for the automatic structured reporting of regional findings in [^123^I]FP-CIT SPECT. However, the CNN-based prediction of the 5-score was correct in all 4 striatal subregions in about 50% of the scans (Table 2). This demonstrates the clinical usefulness of the proposed CNN, given that all datasets employed in this study except the first external dataset were representative of everyday clinical routine.

The specific definition of the “borderline” category was also the rationale for the binary global score derived from the regional 5-scores to require at least “moderate reduction” in at least one putamen to classify the whole scan as reduced, a “borderline” finding was not sufficient. This rule is in line with the fact that the threshold for motor symptoms in PD usually lies between 46 and 64% reduction of the putamen SBR [60]. The binary global score derived from the regional 5-scores achieved ≥ 95% accuracy for the binary scan-level classification (Table 2), well within the range of accuracy estimates reported for direct binary classification of DAT-SPECT by CNN [19–43].

Nevertheless, we do not recommend to replace CNN-based direct binary classification of DAT-SPECT by CNN-based regional categorization, but rather to use both to complement each other. Useful application scenarios are automatic identification of “atypical” patterns, the identification of uncertain cases and assistance in structured reporting of DAT-SPECT. “Atypical” patterns that might be detected automatically by the CNN-based regional categorization include more pronounced reduction in the caudate compared with the putamen or complete “loss” of one striatum, while the other striatum presents with normal tracer uptake. Both patterns might be caused by structural/vascular lesions and, therefore, require careful inspection by the physician, preferably with reference to structural MRI [4]. It is currently unclear how direct CNN-based binary classification of DAT-SPECT is affected by structural/vascular lesions. Some studies on CNN-based binary classification of DAT-SPECT excluded cases with relevant structural/vascular lesions [42], many other studies used data from the PPMI that excluded subjects with “previously obtained MRI scan with evidence of clinically significant neurological disorder” (clinicaltrials.gov/study/NCT04477785). Other studies did not make statements about this point. There are no studies that addressed the impact of structural/vascular lesions on deep learning-based classification of DAT-SPECT to the best of our knowledge. The datasets included in the current study also excluded cases with clearly “atypical” pattern according to visual inspection and, therefore, are not suitable to test the utility of the CNN-based regional analysis for the identification of “atypical” patterns. This should be tested in future studies.

For the identification of uncertain cases, the binary global score derived from the 4 regional 5-level scores would be compared with the direct CNN-based binary scan-level classification. Agreement of the two independent predictions would increase the confidence that the prediction is actually correct. Discrepant predictions would indicate an uncertain case that requires particularly careful visual inspection by an experienced physician. The 96–97% accuracy of the binary global score derived from the regional 5-scores relative to the binary reference standard observed in the two independent test datasets is in line with 5–10% uncertain cases in clinical DAT-SPECT [56, 57] and, therefore, supports the use of CNN-based regional analysis for this task. This, too, should be tested in future studies.

Currently, structured reporting of DAT-SPECT at our site is performed via visual analysis of the images followed by subjective description of the signal in each of the striatal subregions. The proposed tool might be used in such a scenario by suggesting the categorical description to the user and let her/him apply corrections if desired. This procedure has the potential to reduce reading time while increasing the reproducibility. For example, the model correctly predicted the categories in all four regions in about 50% of the images, which suggests that in about half of the scans, no correction by the user is required.

Limitations of the current study include the automatic generation of the regional categorical reference labels based on fitting a Gaussian mixture model to the histogram of regional SBR estimates. However, it is not clear that visual reference labels would be superior, given considerable between- and within-raters variability that is to be expected for a visual 5-score. The three considered “reduced” categories (moderate reduction, strong reduction, almost missing) of the reference standard were separated by the 66th percentile and by the 33th percentile of the “reduced” SBR in order to include about the same number of cases into each of these categories. This can be advantageous from a statistical point of view, but it might not be optimal from a clinical point of view. Transformation of SBR values to z-scores relative to the mean and standard deviation of “non-reduced” SBR and then defining the “reduced” categories based on predefined cutoffs on the z-scores might be more appropriate from a clinical point of view. Quality loss of the automatic reference labels due to the sensitivity of SBR-based measures to site- and camera-specific variability was avoided by fitting the Gaussian mixture model separately for each dataset. A further limitation of the study is that only the coarse anatomical subdivision of the striatum into caudate and putamen was considered. A finer subdivision, for example, into caudate, anterior putamen and posterior putamen [61] or into striatal subregions according to cortico-striatal connections [62, 63], might be tested in further studies. Finally, systematic information on the severity of clinical symptoms (e.g., based on part III of the Unified Parkinson’s disease rating scale) was not available for retrospective analysis in the vast majority of the included patients (most of the patients included in both in-house datasets and in the second external dataset had been referred to [^123^I]FP-CIT SPECT by external neurology practices so that we did not have access to these patients’ files). This prevented us from testing the CNN-based 5-level categorization of specific [^123^I]FP-CIT binding in striatal subregions for association with clinical severity. However, the severity of clinical symptoms in neurodegenerative parkinsonian syndromes in general is not very strongly related to the extent of nigrostriatal degeneration. This is in part due to the high threshold of nigrostriatal degeneration required for parkinsonism to occur. Post-mortem studies have shown that motor symptoms in Parkinson’s disease begin at fairly advanced stages of nigrostriatal degeneration, when the loss of dopamine neurons has reached about 50% [64], the depletion of striatal dopamine has reached about 70% [65], and the loss of DAT in the unilateral posterior putamen has reached about 50% [66]. These post-mortem findings were supported by a [^123^I]FP-CIT SPECT study, which estimated the symptom threshold to be between 46 and 64% reduction in putamen SBR [60]. A recent report of two individual patients with prodromal Parkinson’s disease [67] suggests that treatment with the modified amino acid acetyl-DL-leucine not only improves symptoms but also reverses the loss of striatal DAT as assessed by [^123^I]FP-CIT SPECT. Future studies might test the CNN-based 5-level categorization of specific [^123^I]FP-CIT binding for the monitoring of striatal DAT availability under acetyl-DL-leucine treatment.

In conclusion, automatic scanner-independent CNN-based 5-level categorization of the [^123^I]FP-CIT uptake in striatal subregions is feasible with clinically useful accuracy. It could be used for automatic identification of uncertain borderline cases and “atypical” patterns of striatal [^123^I]FP-CIT uptake, both of which might present an increased risk of false decisions in direct automatic scan-level classification of DAT-SPECT. Furthermore, it could reduce reading time and increase reproducibility in structured reporting of DAT-SPECT.

## Supplementary Information

Below is the link to the electronic supplementary material.

Supplementary file1 (DOCX 485 KB)

## Data Availability

The inference code and the trained network weights are publicly available at https://github.com/ThomasBudd/dat_spect_ud.

## Abbreviations

[^123^I]FP-CIT: N-ω-Fluoropropyl-2β-carbomethoxy-3β-(4-I-123-iodophenyl)nortropane
CNN: Convolutional neural network
DAT: Dopamine transporter
DVR: Distribution volume ratio
IID: Independent identical distribution
OOD: Out-of-distribution
PD: Parkinson’s disease
PPMI: Parkinson’s progression markers initiative
SBR: Specific binding ratio
SPECT: Single-photon emission computed tomography
