# Supplementary material for: Fully automatic categorical analysis of striatal subregions in dopamine transporter SPECT using a convolutional neural network
Source: Ann Nucl Med. 2025 Mar 16;39(6):618–30. doi: 10.1007/s12149-025-02038-3 (PMC12095456; doi:10.1007/s12149-025-02038-3)
Supplement: Supplementary file 1 — Supplementary file1 (DOCX 485 KB) [file 12149_2025_2038_MOESM1_ESM.docx]

**Supplementary Material**

**Supplementary Tab. 1** SPECT acquisition parameters in the first in-house dataset. (LEHR = low-energy-high-resolution collimator, LEHRHS = low-energy-high-resolution-high-sensitivity collimator)

|  | Siemens e.cam with LEHR | Siemens Symbia TruePoint with LEHR | Siemens Symbia TruePoint with fan-beam | Mediso AnyScan Trio with LEHRHS (dual-head mode) |
| --- | --- | --- | --- | --- |
| # Scans | 704 | 147 | 457 | 432 |
| # Views | 128 | 120 | 120 | 120 |
| Scan arc [°] | 180 | 180 | 180 | 180 |
| Angular step [°] | 2.81 | 3 | 3 | 3 |
| Radius of rotation [mm] | 165±18 | 166±19 | 153±13 | 146±12 |
| Matrix size | 128x128 | 128x128 | 128x128 | 128x128 |
| Pixel size [mm^2^] | 4.80x4.80 | 3.90x3.90 | 3.90x3.90 | 2.43x2.43 |
| Energy window [keV] | 147-171 | 147-171 | 147-171 | 143-175 |
| Total net scan duration [min] | 32-41 | 30-40 | 40 | 40 |

**Supplementary Tab. 2** Results of the Gaussian mixture model fit of the SBR histograms and resulting thresholds for the separation of the 5 regional categories. All values are given for putamen/caudate respectively.

|  | In-house 1 | In-house 2 | External 1 | External 2 |
| --- | --- | --- | --- | --- |
| Mean „non-reduced“ (M_1_) | 1.615/2.631 | 1.769/2.829 | 1.100/1.691 | 1.514/2.465 |
| SD „non-reduced“ (SD_1_) | 0.345/0.542 | 0.413/0.777 | 0.348/0.510 | 0.327/0.475 |
| Mean „reduced“ (M_2_) | 0.636/1.545 | 0.546/1.580 | 0.486/1.156 | 0.622/1.421 |
| SD „reduced“ (SD_2_) | 0.185/0.458 | 0.251/0.463 | 0.142/0.274 | 0.186/0.422 |
| Threshold normal vs. borderline | 1.175/2.179 | 1.230/2.190 | 0.872/1.530 | 1.093/2.018 |
| Threshold borderline vs. moderate reduction | 0.978/2.042 | 1.008/2.046 | 0.796/1.463 | 0.946/1.912 |
| Threshold moderate vs. strong reduction | 0.716/1.729 | 0.651/1.688 | 0.577/1.251 | 0.705/1.591 |
| Threshold strong reduction vs. (almost) missing | 0.558/1.389 | 0.445/1.362 | 0.441/1.023 | 0.547/1.262 |


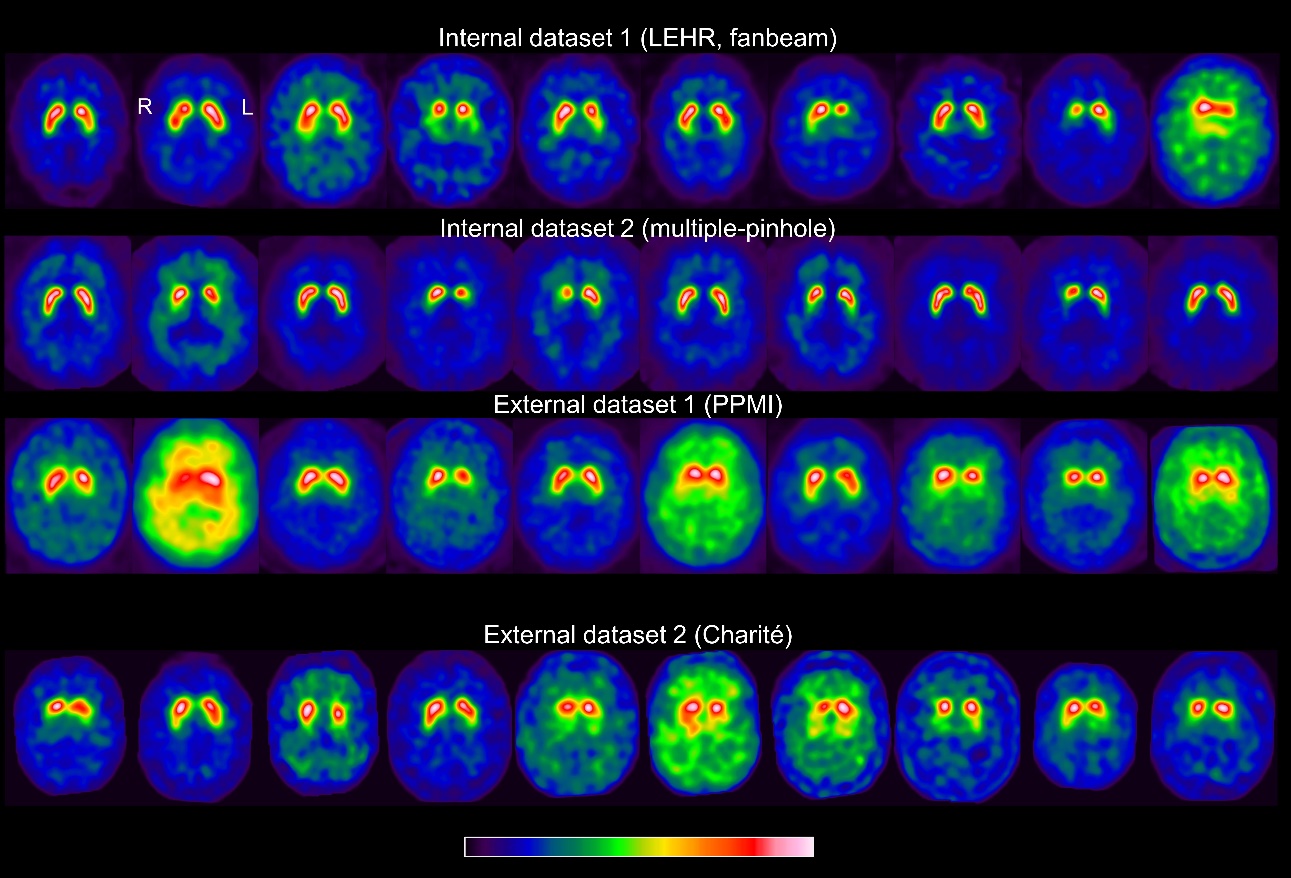


**Supplementary Fig. 1** Ten randomly selected images from each of the 4 datasets.


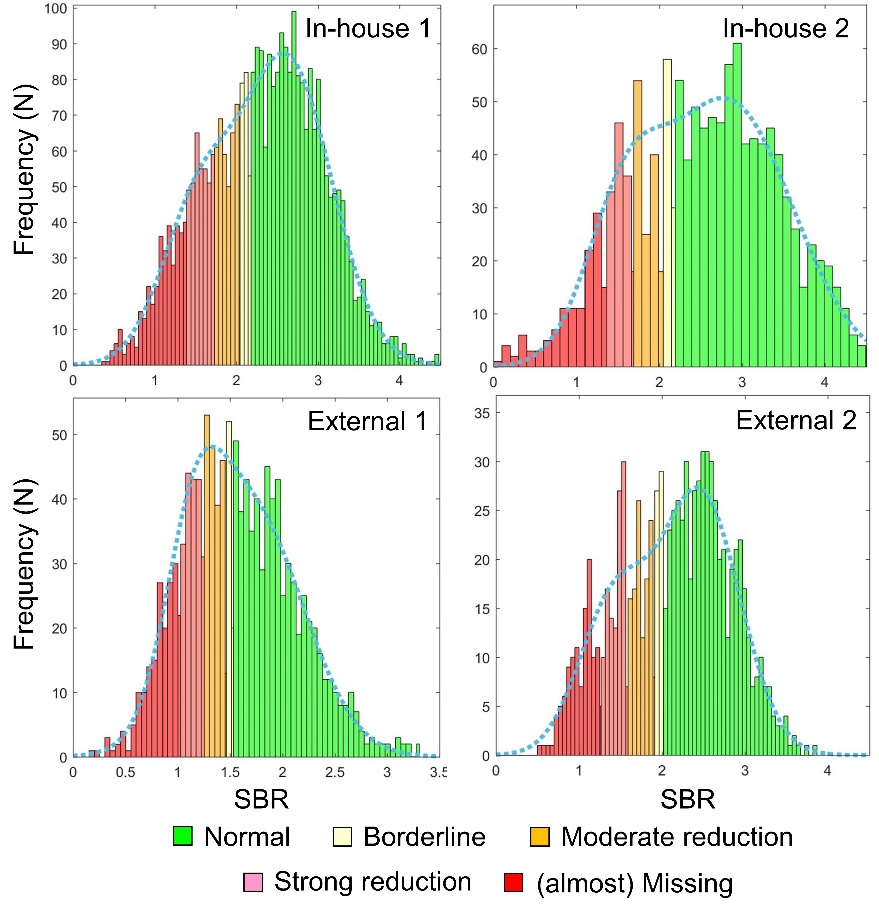


**Supplementary Fig. 2** Automatic generation of the reference standard for the categorical 5-level score in the caudate based on the fit of a Gaussian mixture model to the histogram of the caudate specific binding ratio (SBR). This was performed separately for each dataset.
